# GREENSPACE AND COGNITION: EXPLORING SPECIFIC GREENSPACE MEASURES

**DOI:** 10.1093/geroni/igac059.3087

**Published:** 2022-12-20

**Authors:** Aniruddh Ajith, Sara Godina, Xiaonan Zhu, Alyson Harding, Andrea Rosso

**Affiliations:** University of Pittsburgh, Pittsburgh, Pennsylvania, United States; University of Pittsburgh, Pittsburgh, Pennsylvania, United States; University of Pittsburgh, Pittsburgh, Pennsylvania, United States; University of Minnesota School of Public Health, Minneapolis, Minnesota, United States; University of Pittsburgh, Pittsburgh, Pennsylvania, United States

## Abstract

Increased exposure to greenspace is potentially associated with lower risk of Alzheimer’s disease and related disorders (ADRD) through increased physical activity, decreased stress, cleaner air, and social cohesion. Previous studies have primarily used global measures of greenspace, thus specific aspects of different types of greenspaces are overlooked. We assess how different measures of greenspaces associate with global cognitive functioning. We examined data from year 10 of the Pittsburgh site of Health, Aging, and Body Composition Study, a longitudinal cohort study of community-dwelling, Black and White adults aged 70–79 years at baseline. Measures derived from the National Land Cover Database (NLCD) use satellite imaging and include total greenspace, forests, and an index of greenspace diversity. Measures from Google Street View virtual neighborhood audits include dedicated greenspace that promotes physical activity, abandoned buildings, and undeveloped land. We measured global cognitive functioning using the Modified Mini-Mental State Examination (3MS). We conducted cross-sectional linear regression models. Covariates included race, education, and neighborhood socioeconomic status. Age was not included as a covariate because the range was narrow, and results did not vary by age. The sample (n=584) was 82.2 years old on average, 57% female, and 35% Black. Total greenspace (F=0.46, p=0.49), forests, and greenspace diversity, dedicated greenspace for physical activity (F=0.87, p=0.35), undeveloped land (F=1.49, p=0.22), and abandoned buildings (F=3.6, p=0.058) were not associated with 3MS, although the relation with abandoned buildings approached significance. We found no association between greenspaces and ADRD, although specific measures, such as abandoned buildings, may need further investigation.

